# Translocation of bacterial NOD2 agonist and its link with inflammation

**DOI:** 10.1186/cc7980

**Published:** 2009-07-28

**Authors:** Oh Yoen Kim, Antoine Monsel, Michèle Bertrand, Jean-Marc Cavaillon, Pierre Coriat, Minou Adib-Conquy

**Affiliations:** 1 Cytokines & Inflammation Unit, Institut Pasteur, 28 rue Dr. Roux, 75015 Paris, France; 2 Department of Anesthesiology and Critical Care, Université Pierre et Marie Curie – Paris 6, and Centre Hospitalier Universitaire Pitié-Salpêtrière, Assistance-Publique Hôpitaux de Paris, Paris, France

## Abstract

**Introduction:**

The gut is often considered as the motor of critical illness through bacterial translocation, which amplifies the inflammatory response and alters the immune status. However, systemic bacterial translocation was rarely proven and endotoxin measurement only reflects translocation of Gram-negative-derived products. The process could be more frequently identified if peptidoglycan, derived from both Gram-negative and Gram-positive bacteria, was measured.

**Methods:**

We developed a new tool to detect circulating peptidoglycan-like structure using a NOD2-transfected cell line. We also measured plasma and cell-associated endotoxin and different plasma markers of inflammation. We studied 21 patients undergoing abdominal aortic surgery (AAS), and 21 patients undergoing carotid artery surgery (CAS) were included as negative controls. Patients were sampled during surgery until two days post-surgery.

**Results:**

In 90.5% of the AAS patients, a NOD2 agonist peak was detected in plasma before aortic clamping, but after gut manipulation by the surgeon, and persisted after blood reperfusion. As expected, no peak was detected in plasma from CAS patients (*P *= 0.003). Leukocyte-bound endotoxin appeared after blood reperfusion in 71% of the AAS patients, and circulating endotoxin was detected for 57% of them. The levels of interleukin (IL)-6, IL-10 and of inflammatory markers (C-reactive protein, procalcitonin) were maximal at postoperative day 1 or 2 in AAS patients. The levels of circulating NOD2 agonist positively correlated with those of cortisol and IL-10.

**Conclusions:**

The measurement of circulating NOD2 agonist gives a higher informative tool than that of circulating endotoxin for early and sensitive detection of the translocation of bacterial products. The data suggest that circulating NOD2 agonist contributes to further enhance the stress response following surgery.

## Introduction

The gut has often been claimed to be the motor of critical illness [1]. Translocation of microbial products has been reported in different clinical settings such as in patients with pancreatitis [2], cirrhosis [3], edema secondary to congestive heart failure [4], chronic HIV infection [5], after cardio-pulmonary bypass [6], after hemorrhagic shock [7], in patients resuscitated after cardiac arrest [8], and after abdominal aortic surgery [9].

Endotoxin (lipopolysaccharide (LPS)) is a microbial product commonly measured in the bloodstream, and its levels correlate with survival in patients with sepsis [10]. Levels of circulating endotoxin were also shown to correlate with liver function deterioration in patients with cirrhosis [11] or with the occurrence of multiorgan failure in intensive care unit patients [12]. Although the occurrence of endotoxinemia is more frequent than positive hemocultures, endotoxin being present only in Gram-negative bacteria, its measurement does not reflect the translocation of Gram-positive bacteria-derived compounds [13]. Furthermore, the measurement of LPS in plasma is difficult because of the presence of many interfering molecules such as soluble CD14, LPS-binding protein, and high-density lipoproteins [14-16]. LPS may also be trapped by circulating cells carrying receptors for LPS, such as monocytes. For example, during meningococcal infection, leukocyte-bound LPS was found in all studied patients, whereas circulating endotoxin was detected in only two out of five patients [17].

On the other hand, peptidoglycan (PGN) is a component of both Gram-positive and Gram-negative bacterial cell walls and its levels in plasma may better reflect bacterial translocation, as found in 10 patients undergoing cardio-pulmonary bypass [18]. However, the assay used in this study was not specific for bacterial products and also measured fungal components such as β-glucan. Recent studies reported that PGN and its fragments are recognized by intracellular pattern-recognition molecules, members of the nucleotide-binding oligomerization domain (NOD) family [19]. In particular, NOD2 recognizes a PGN motif present on both Gram-positive and Gram-negative bacteria. This sensing initiates an intracellular cascade that leads to the activation of the nuclear transcription factor NF-κB and an inflammatory process [20,21]. Using this information, we developed a new tool to detect circulating PGN-like structures using a NOD2-transfected cell line and the luciferase reporter gene [22].

Vascular surgery like all other surgery is associated with an inflammatory process and an alteration of the immune status that may favor the occurrence of nosocomial infections [23-26]. Endotoxin translocation was previously reported in some patients after abdominal aortic surgery (AAS), associated with manipulation of the gut and aortic clamping [9], leading to a significant decrease in mesenteric blood flow and the subsequent alteration of oxygen delivery to the intestinal epithelial carriers [27,28]. The translocation could further amplify the inflammatory response and alter the immune status, and may contribute to the development of postoperative complications [29-32]. Therefore, we aimed to detect circulating NOD2 agonist in AAS patients susceptible to bacterial translocation, to determine its frequency and its kinetics. Patients undergoing carotid artery surgery (CAS) were included as a control group. In addition, we analyze leukocyte-bound LPS, and measured C-reactive protein (CRP), procalcitonin (PCT) and cortisol, as well as several pro- and anti-inflammatory cytokines to assess the level of systemic inflammation in the two groups of patients.

## Materials and methods

### Subjects and operation

After approval of the study by the Ethics Committee for Human Research of Pitié-Salpêtrière Hospital (Session of 4 April, 2007), patients scheduled for AAS were included in this prospective observational study from June 2007 to April 2008 (n = 21). As a control group, patients scheduled for CAS were also included (n = 21). Excluded from the study were patients: undergoing celoscopic surgery or surgery on the thoracic aorta, with signs of pre-operative infection, undergoing chronic dialysis, under anti-inflammatory medication or antibiotics treatment before surgery, presenting with on-going or neoplasic hematologic pathology, and who were immunosuppressed. All patients gave informed consent. The protocol followed for preoperative medication and anesthesia was similar in both groups of patients. The only difference was that treatment with anti-platelet aggregation agents was discontinued five days before surgery for AAS patients, whereas it was continued until the day of surgery for CAS patients.

Usual premedication was maintained except for angiotensin-converting enzyme inhibitors and angiotensin II antagonists, which were discontinued the day before surgery. All patients were premedicated with midazolam 5 mg given orally one hour before surgery. During the operative period, all patients were anesthetized by target-controlled infusion of propofol, sufentanil, and cisatracurium. Antibioprophylaxis was performed using cefamandole. Depending on hemodynamics and hematocrit, fluid loading was performed using crystalloid infusion (Ringer's lactate or isotonic saline) and colloid infusion (hydroxyethylstach 130/0.4), associated with blood transfusion, if necessary, to maintain a hemoglobin level above 10 g/dl. Approximately 30 minutes before the end of surgery, all patients received paracetamol for postoperative analgesia, and in recovery room received intravenous morphine until pain relief was achieved.

### Blood sampling

Blood samples were collected into the sodium citrated vacuum tubes as follows: immediately before anesthesia induction (T_1_); before incision (T_2_), before aortic clamping (AAS patients) or carotid artery clamping (CAS patients; T_3_) and after blood reperfusion (T_4_) during the surgery, and on postoperative day one (POD1) and two (POD2) after the surgery. One 5 ml tube was immediately centrifuged and plasma samples were stored at -80°C until assayed (within two months). Another 5 ml tube was used for the analysis of leukocyte-bound LPS.

### Isolation of peripheral blood mononuclear cells from whole blood

For measurement of endotoxin associated with circulating leukocytes, peripheral blood mononuclear cells (PBMC) were isolated from whole blood after dilution 1:1 with RPMI-1640 (Lonza, Verviers, Belgium) and centrifugation (680 *g*, 15°C for 20 minutes) on Ficoll-Hypaque (Eurobio, Les Ulis, France). After centrifugation, the cells at the medium/Ficoll interface were collected, washed with RPMI-1640 and centrifuged (350 *g*, 10°C for five minutes). The pellet was resuspended with sterile endotoxin-free saline (0.9% sodium chloride) (Fresenius, Sèvres, France). PBMC were lysed by five cycles of freezing and thawing, and stored at -80°C until assayed (within two months).

### Detection of circulating NOD2 agonist in human plasma

Human embryonic kidney (HEK) 293T cells (ATCC, Manassas, VA, USA) were cultured in Dulbecco's modified Eagle's medium (Invitrogen, Carlsbad, CA, USA) supplemented with 10% fetal calf serum (PAA, Pasching, Austria). HEK293T cells were seeded into 24-well plates at a density of 10^5 ^cells/ml (500 μl/well). The detection of NOD2 agonist in biologic fluids using this transfected cell line has been previously described [22]. Briefly, HEK293T cells were transfected with a plasmid permitting the constitutive expression of NOD2 (sensor of both Gram-positive and Gram-negative PGN) and an NF-κB-dependent reporter gene coding for luciferase. To the transfected cells, 50 μl of plasma was added and then incubated for six hours. The plasma were either from patients or from healthy volunteers (ICAReB, Institut Pasteur, Paris, France). The presence of NOD2 agonist in plasma was assessed by luciferase activity in cell lysates. To the cells, 100 μl of lysis buffer (25 mM Tris-phosphate pH 8, 8 mM MgCl_2_, 1 mM dithiothreitol, 15% glycerol and 1% Triton X-100) was added and luciferase activity in 10 μl of cell extracts was measured in a microplate luminometer (LB960 luminometer centro, Berthold Technologies, Germany) after the addition of 100 μl of substrate buffer to a final concentration of 1.8 mM luciferin and 1 mM ATP. Luciferase activity was expressed as relative light unit. In some experiments, a frameshift mutant of NOD2 (fsNOD2) devoid of NF-κB activation capacity in response to NOD2 ligand was used as a negative control (kind gift of Dana J Philpott, University of Toronto, Toronto, Canada). As positive controls, we used PGN from *Staphylococcus aureus *(InvivoGen, San Diego, CA, USA), *Escherichia coli *(kind gift of Dominique Mengin-Lecreux, Orsay, Paris XI University France), as well as anaerobic Gram-positive (*Clostridium clostridioforme*) and Gram-negative (*Bacteroides thetaitamicron*) bacterial PGN (kind gift of Michel Popoff, Institut Pasteur and Dominique Mengin-Lecreux, Orsay, Paris XI University France). In addition, muramyl dipeptide (MDP), TNF-α and IL-1β (R&D system, Minneapolis, MN, USA) were also tested for comparing the specificity between NOD2 and fsNOD2 transfected systems. The results from study subjects were presented as fold change of luciferase activity based on the initial values (T_1_, before anesthesia). Detection limit was calculated based on the mean value ± two standard deviations (SD) of the fold values at T_2 _in control subjects. Each test was performed in triplicates. A fold increase of 1.57 was considered as positive.

### Measurement of endotoxin in peripheral blood mononuclear cells (PMBC) and plasma

Detection of bacterial endotoxin in plasma is hindered by the yellow color of plasma and the presence of inhibitors. We avoided the background absorbance of plasma by using a diazo-coupling limulus amebocyte lysate (LAL) assay that gives a magenta coloration (detection limit: 1.6 pg/ml) (Associates of Cape Cod, East Falmouth, MA, USA), and by heating plasma at 65°C for 30 minutes to inactivate inhibitors as described [8]. Endotoxins from lysates of PBMC were assayed with QCL-1000 Chromogenic LAL kit (detection limit: 5 pg/ml) (Lonza, Walkersville, MN, USA). The assays were carried out according to the manufacturers' instructions. Samples were tested in duplicate.

### Measurement of cytokines and cortisol

IL-6 and IL-10 were measured in plasma samples by ELISA (DuoSet, R&D Systems, Minneapolis, MN, USA). Plasma levels of cortisol were measured using an enzyme immunoassay (AbCys, Paris, France). The assays were carried out according to the manufacturer's instruction. The color reaction was read at 450 nm using a MRXELISA microplate reader (DYNEX, Gaithersburg, PA, USA).

### Leukocyte count and measurement of C-reactive protein and procalcitonin

Leukocyte counts (per mm^3^) in whole blood and the measurement of plasma levels of CRP (MODULAR assay, Roche, Meylan, France) and PCT (Kryptor assay, BRAHMS, Clichy, France) were routinely performed at time points T_1_, T_4_, POD1, and POD2 in the hospital.

### Statistical analysis

Data were expressed as median (interquartile range) or mean ± standard error of the mean (SEM), as indicated. The value zero was assigned to values less than the assay detection limit. General characteristics of the two surgery groups (AAS versus CAS) were tested by the Mann-Whitney U test, or Fischer's exact test depending on the data. Circulating levels of NOD2 agonist, endotoxin, and inflammatory markers before, during, and after the surgery of the two groups were examined by the repeated measure one-way analysis of variance followed by the least significant difference method. The relation between two continuous variables were evaluated using Spearman's rank correlation tests. *P*-values less than 0.05 were considered to be significant. All statistical analyses were performed with SPSS version 12.0 for Windows (Statistical Package for the Social Science, SPSS, Chicago, IL, USA).

## Results

### Set-up of the NOD2 agonist detection system

**Figure 1:**
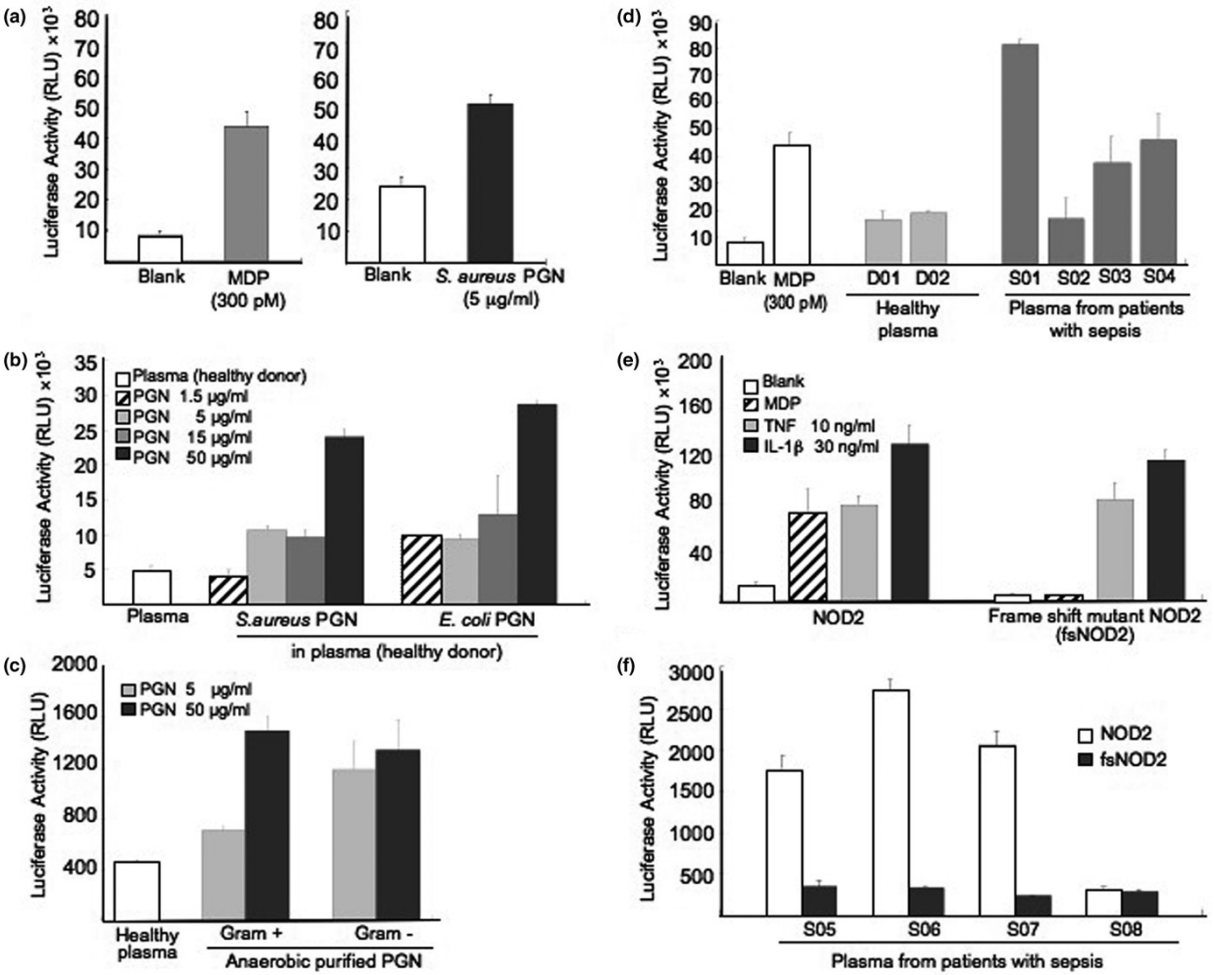
Detection of circulating peptidoglycan using NOD2 transfected cell line and NF-κB-luciferase reporter gene. **(a) **Activation with muramyl dipeptide (MDP) or peptidoglycan (PGN) from *Staphylococcus aureus *(*S. aureus*). Human embryonic kidney (HEK) 293T cells transfected with nucleotide-binding oligomerization domain (NOD) 2 and nuclear factor (NF)-κB luciferase expression plasmids were stimulated with MDP or *S. aureus *PGN. After six hours of incubation, luciferase activity in cell extracts was measured and expressed as relative light unit (RLU). The figure is the mean ± standard error of the mean (SEM) of three independent experiments performed in triplicates. **(b) **Sensitivity of NOD2-detection of both Gram-negative and Gram-positive bacterial PGN added in healthy human plasma. HEK293T cells transfected with NOD2 and NF-κB luciferase expression plasmids were stimulated with various concentrations of PGN from *S. aureus *or *Escherichia coli *diluted in plasma from healthy controls. The figure is the mean ± SEM of three independent experiments performed in triplicates. **(c) **NOD2-detection of both purified and non-purified anaerobic Gram-negative and Gram-positive bacterial PGN incubated in healthy human plasma. HEK293T cells transfected with NOD2 and NF-κB luciferase expression plasmids were stimulated with purified or non-purified anaerobic bacterial PGN from Gram-positive (*Clostridium clostridioforme*) and Gram-negative (*Bacteroides thetaiotamicron*) diluted in plasma from healthy controls. The figure is the mean ± SEM of three independent experiments performed in triplicates. **(d) **Positive signals in plasma from septic patients. HEK293T cells transfected with NOD2 and NF-κB luciferase expression plasmids were stimulated with plasma from healthy controls and sepsis patients. As a positive control, MDP was used. The figure is the mean ± SEM of three independent experiments performed in triplicates. **(e) **Specificity of NOD2 detection of bacterial PGN fragment in comparison with a frame shift mutant of NOD2 (fsNOD2) transfected system. HEK293T cells, transfected with NOD2 or fsNOD2 and NF-κB luciferase expression plasmids, were stimulated with MDP, TNF-α or IL-1β. The figure is the mean ± SEM of three independent experiments performed in triplicates. **(f) **Comparison of the signals between NOD2 and fsNOD2 in septic plasma. HEK293T cells transfected with NOD2 or fsNOD2 and NF-κB luciferase expression plasmids were stimulated with plasma samples from sepsis patients. The figure is the mean ± SEM of three independent experiments performed in triplicates.

To develop a new tool for the detection of circulating NOD2 agonist, we used the HEK293T cell line constitutively expressing NOD2 as a sensor of bacterial PGN through its minimal motif MDP [20,21]. The cell line was transfected with a luciferase reporter gene under the control of an NF-κB-dependent promoter. This system was able to give positive signals when simulated with MDP (Figure 1a, left panel) or PGN from *S. aureus *(Figure 1a, right panel). The detection system was also functional when PGN was added to healthy donor's plasma (Figure 1b). The system efficiently detected various concentrations of PGN from *S. aureus *(Gram-positive bacteria) or *E. coli *(Gram-negative bacteria) incubated in plasma from healthy donors.

Gut flora is mainly composed of anaerobic bacteria, and the detection of anaerobic bacterial PGN may be more relevant for bacterial translocation. Thus, we also checked that our system was responsive to the stimulation with purified anaerobic Gram-positive as well as Gram-negative bacterial PGN incubated in healthy plasma (Figure 1c). NF-κB activation was also obtained with plasma samples from sepsis patients, used as positive controls, as compared with those from healthy donors (Figure 1d). The specificity of our system was checked by transfecting an expression plasmid for fsNOD2, a mutant unable to activate NF-κB in response to MDP (Figure 1e). HEK293T cells express TNF and IL-1 receptors. Accordingly, NF-κB activation in response to TNF or IL-1β, turned on the luciferase gene expression and cells transfected with NOD2 or fsNOD2 were similarly responsive to these inflammatory cytokines. Plasma samples from sepsis patients (defined according to Bone and colleagues [33]), showed a positive signal in the NOD2 transfected system, but did not induce activation in the fsNOD2-transfected system (Figure 1f). This latter observation indicates that the putative inflammatory cytokines present in the plasma of sepsis patients are not in sufficient amounts to activate the luciferase gene, and that the NOD2 transfection system can selectively and specifically detect bacterial NOD2 agonists.

### Patients' characteristics

Table 1 presents general characteristics of the study subjects and information about the operation and postoperative complications. There was no significant difference in basic characteristics such as body weight, proportions of gender, cigarette smoking, and medical history between the two surgery groups, although CAS patients tended to be older. Medications were not significantly different between the two groups, except for the use of anti-platelet aggregation agents. Durations of operation and vascular clamping were longer in AAS patients than in CAS patients. The amounts of blood loss and blood transfusion during surgery were also higher in AAS patients, who had longer durations of mechanical ventilation and hospitalization than CAS patients.

**Table 1:** Demographic data and information for the operation and postoperative complications of study subjects

	Median (min to max), or n (%)		
	AAS (n = 21)	CAS (n = 21)	*P *value
Age (years)	67 (46 to 85)	77 (42 to 88)	0.06
Body weight (kg)	73 (51 to 97)	75 (50 to 95)	NS (0.70)
Male	15 (85.7)	18 (71.4)	NS (0.45)
Cigarette smoking	20 (95.2)	17 (81)	NS (0.34)
Medical history			
Coronaropathy	7 (33.3)	5 (23.8)	NS (0.50)
Angina pectoris	1 (4.8)	5 (23.8)	
Myocardial infarction	2 (9.5)	2 (9.5)	
Coronary bypass	1 (4.8)	3 (14.3)	
Transluminal angioplasty	2 (9.5)	2 (9.5)	
Hypertension	15 (71.4)	18 (85.7)	NS (0.45)
Heart failure (left ventricular)	1 (4.8)	2 (9.5)	NS (1.00)
Diabetes mellitus	5 (23.8)	5 (23.8)	NS (1.00)
Chronic bronchial obstruction	4 (19)	7 (38.1)	NS (0.29)
Renal insufficiency	7 (33.3)	8 (38.1)	NS (0.75)
Medications			
Antiplatelets agents	12 (60)	21 (100)	0.001
Statins	9 (42.9)	15 (71.4)	0.060
Operation information			
Blood loss (mL)	1000 (400 to 3500)	100 (50 to 900)	< 10^-4^
Fluid infusion (mL)	4500 (3000 to 9000)	1500 (1000 to 3000)	< 10^-4^
Blood transfusion			
Red blood cells	1 (0 to 6)	0 (0 to 2)	< 10^-4^
Fresh frozen plasma	1 (0 to 4)	0	NS (0.15)
Cell-saver	2 (0 to 8)	0	NS (1.00)
Operation duration (hours)	2.5 (1.6 to 6.5)	1.2 (1 to 2.5)	< 10^-4^
Vascular clamping duration (min)	50.5 (14 to 90)	26 (12 to 45)	< 10^-4^
Catecholamines	2 (9.5)	0	0.5
Postoperative complication, n (%)	11 (52.4)	6 (28.0)	0.02
Cardiac complication	6 (28.6)	1 (4.8)	NS (0.09)
Respiratory complication	6 (28.6)	1 (4.8)	NS (0.09)
Infection	4 (19)	2 (9.5)	NS (0.70)
Hemorrhage	1 (4.8)	0	NS (1.00)
Reoperation	3 (14.3)	0	NS (0.23)
Other (pancreatitis, etc)	3 (14.3)	2 (9.5)	NS (1.00)

Continuous values were compared by the Mann-Whitney test and frequencies or percentages were tested by the Chi-squared or Fisher's exact test.

AAS = abdominal aortic surgery group; CAS = carotid artery surgery; NS = statistically no significance between the two groups.

Eleven of 21 AAS patients and 6 of 21 CAS patients had postoperative complications (*P *= 0.02). Among them, four AAS patients and three CAS patients were diagnosed as having infectious complications, but only one of the four AAS patients was diagnosed within the observational period (at POD2). In addition, four other AAS patients showed a systemic inflammatory response syndrome (SIRS), as defined by Bone and colleagues [33], at T_4 _or POD1.

### Detection of bacterial NOD2 agonist in plasma samples from AAS and CAS patients

**Figure 2:**
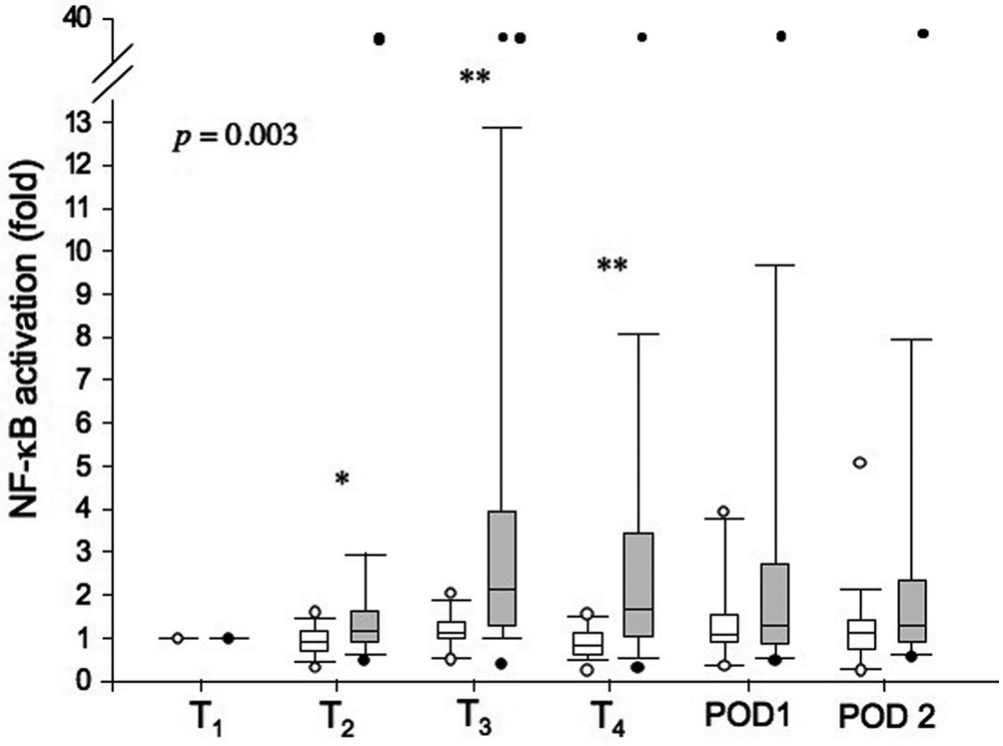
Assessment of bacterial NOD2 agonist levels in the plasma of AAS and CAS patients. Human embryonic kidney (HEK) 293T cells transfected with nucleotide-binding oligomerization domain (NOD) 2 and nuclear factor (NF)-κB luciferase expression plasmids were stimulated with plasma from abdominal aortic surgery (AAS) and carotid artery surgery (CAS) patients. After six hours of incubation, luciferase activity in cell extracts was measured, and NF-κB activation was calculated as fold induction with respect to time point 1 (T_1_). T_1 _(before anesthesia), T_2 _(before incision), T_3 _(before clamping), T_4 _(after blood reperfusion), POD1 (postoperative day 1), and POD2 (postoperative day 2). Data are shown as median and interquartile range. The two groups were compared using analysis of variance repeated measurement and least significant difference. *P*-value (*P *= 0.003) indicates the significant difference between the two patient groups in whole study time. * *P *< 0.05 and **P < 0.01 are from the comparison of the two groups at each time point (Mann-Whitney U test). CAS = white boxes; AAS = grey boxes. Black and white circles (T_2 _to POD2) indicate values within 5th percentile and out of 95th percentile, respectively.

Bacterial NOD2 agonist was measured in patients' plasma samples by the test developed in our laboratory [22]. This *in vitro *test was able to detect specifically PGN from Gram-positive/Gram-negative, aerobic/anaerobic bacteria as shown above. Figure 2 presents the results for the detection of circulating NOD2 agonist during and after surgery in the two groups. Values are provided as fold change of luciferase activity as compared with NF-κB activation before anesthesia (T_1_). NOD2 agonist was detected in the plasma of 90.5% of AAS patients, with a peak occurring before aortic clamping (T_3_). NOD2 agonist levels were still high after blood reperfusion (T_4_), and then gradually declined at POD1 and POD2.

In contrast, no major increase occurred in the plasma of CAS patients. NOD2 agonist was detected in the plasma of 23.8% of these patients during surgery, but the levels were significantly lower than in AAS patients. One AAS patient, who developed SIRS at POD1, had very high levels of NOD2 agonist (20 to 40 fold increase of NF-κB activation) during the whole observational period starting at time point T_2_, which led to a statistically significant difference in the values between the two groups at T_2_. Before surgery, this patient already had large and numerous calcified atheromas in the aorta and other arteries throughout the body, which may be responsible for increased vascular fragility and/or increased ischemia, even before gut manipulation and clamping. If this patient was removed from the analysis, there would be no significant differences between the two groups at time point T_2_, but there were still significant differences in NF-κB activation between AAS and CAS patients before aortic clamping (T_3_) and after blood reperfusion (T_4_).

### Endotoxin levels bound to PBMC and in plasma

**Figure 3:**
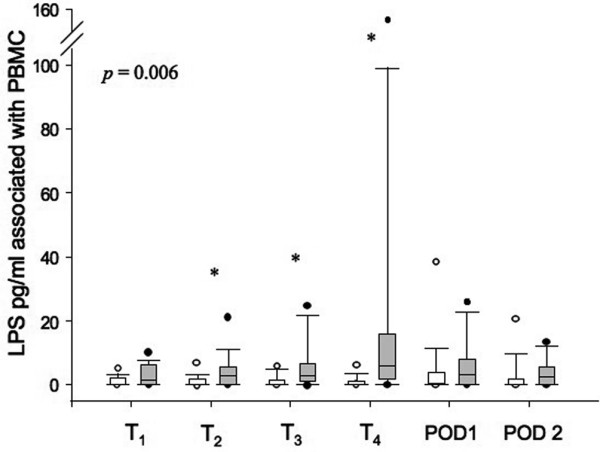
Assessment of endotoxin (LPS) associated with circulating peripheral blood mononuclear cells (PBMC) in AAS and CAS patients. Endotoxin associated with patients' PBMC was measured using a *Limulus *amebocyte assay. T_1 _(before anesthesia), T_2 _(before incision), T_3 _(before clamping), T_4 _(after blood reperfusion), POD1 (postoperative day 1), and POD2 (postoperative day 2). Data are shown as median and interquartile range. The two groups were compared using analysis of variance repeated measurement and least significant difference. *P*-value (*P *= 0.006) indicates the significant difference between the two patient groups in whole time measured. * *P *< 0.05 are from the comparison of the two groups at each time point (Mann-Whitney U test). CAS = carotid artery surgery (white boxes); AAS = abdominal aortic surgery (grey boxes). Black and white circles (T_1 _to POD2) indicate values within 5th percentile and out of 95th percentile, respectively.

The levels of endotoxin in lysates of PBMC are presented in Figure 3. LPS was detected in PBMC lysates of 71.4% of AAS patients, with levels significantly higher than in CAS patients. Highest levels of LPS were observed after blood reperfusion (T_4_) in AAS patients and decreased progressively at POD1 and POD2. On the other hand, the CAS group showed no significant presence of LPS in their PBMC during the observational period. Plasma levels of LPS were also measured in AAS patients. Only 57.1% of the plasmas were positive and the detected levels were much lower than those found in PBMC (data not shown).

### Comparison of the reliability of the assays for the detection of NOD2 agonist and endotoxin

In order to examine the reliability of detection of the NOD2 agonist and endotoxin tests, we compared the positive responses to the tests based on the detection limits. The detection limits of the LAL test in PBMC and plasma were 5 pg/ml and 1.6 pg/ml, respectively. The natural structure derived from PGN following translocation is unknown and may be different from that resulting from enzymatic digestion and purification performed *in vitro*. We avoided extrapolating the levels of luciferase activity to a standard curve of biochemically purified PGN. Our test was expressed as fold increase as compared with the plasma before surgery, each patient being his own control. The detection limit of the NOD2 agonist test showed a 1.57-fold increase in luciferase activity as compared with the value at T_1 _(before anesthesia). This detection limit was calculated on the basis of the mean ± 2SD of the fold increase at T_2 _in control patients. For reference, the mean ± SEM and median fold increase for various PGN (5.0 μg/ml) incubated in healthy plasma were 1.76 ± 0.19 and 1.68, respectively (Figure 1b).

**Figure 4:**
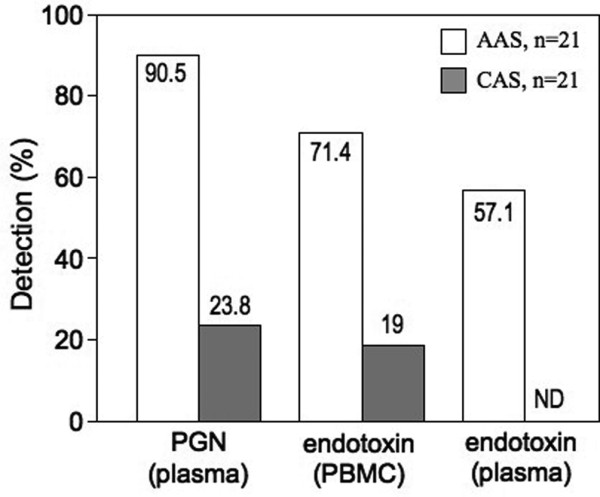
Reliability of the tests for circulating NOD2 agonist in plasma and endotoxin in plasma and associated with PBMC. The figure indicates the percentage (%) of positive patients based on the detection limits of the tests. AAS = abdominal aortic surgery; CAS = carotid artery surgery; NOD = nucleotide-binding oligomerization domain; PBMC = peripheral blood mononuclear cells; PGN = peptidoglycan.

In all AAS and CAS patients, we compared the detection reliability for the NOD2 agonist test in plasma with the endotoxin test in PBMC. In AAS patients, 90.5% (19 out of 21 patients) were positive in the NOD2 agonist test, and 71.4% (15 out of 21 patients) were positive in the LAL test (PBMC-associated LPS). In CAS patients, 23.8% (5 out of 21 patients) were positive in the NOD2 agonist test, and 19% (4 out of 21 patients) in the LAL test (Figure 4). From this result, we can conclude that the NOD2 agonist detection test was more reliable than the endotoxin test.

### Plasma levels of cytokines, CRP, PCT, cortisol and leukocyte counts

**Figure 5:**
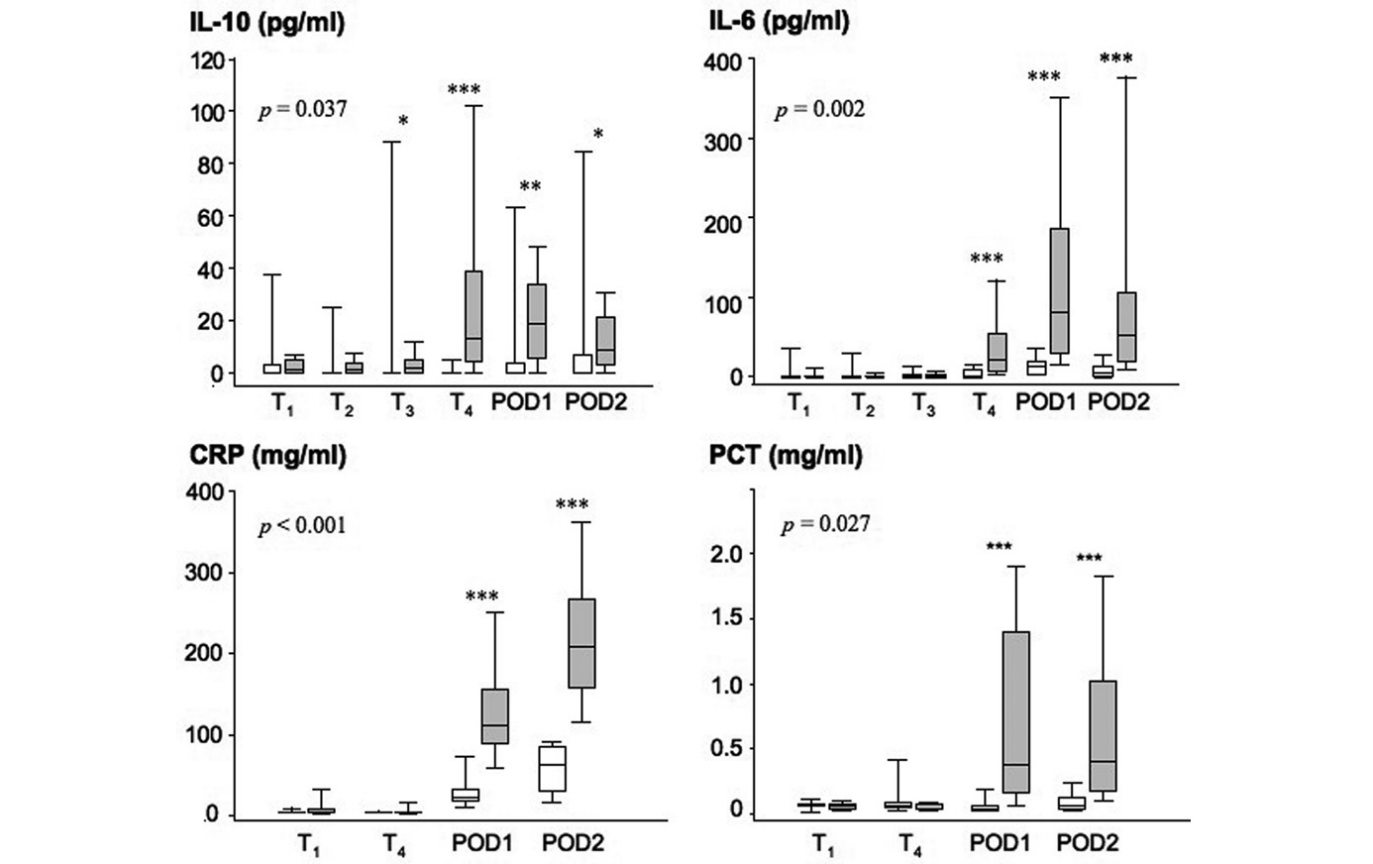
Plasma levels of IL-10, IL-6, C-reactive protein and procalciton in in AAS and CAS patients. Plasma levels of cytokines, C-reactive protein (CRP), and procalcitonin (PCT) were measured at T_1 _(before anesthesia), T_2 _(before incision), T_3 _(before clamping), T_4 _(after blood reperfusion), POD1 (postoperative day 1), and POD2 (postoperative day 2). Data are shown as median and interquartile range. The two groups were compared using analysis of variance repeated measurement and least significant difference. *P*-values indicate the significant difference between the two patient groups at all times measured. * *P *< 0.05, **P < 0.01 and ***P < 0.001 are from the comparison of the two groups at each time point (Mann-Whitney U test). CAS = carotid artery surgery (white boxes); AAS = abdominal aortic surgery (grey boxes).

In order to monitor the inflammatory process, IL-6, IL-10, CRP, PCT, and cortisol were assayed in plasma and leukocytes counted at various time points. As shown in Figure 5, significantly higher amounts of IL-6 and IL-10 were detected in the plasma of AAS patients as compared with CAS. IL-6 was detected in AAS plasma at T_4 _and the highest value was attained at POD1. The presence of IL-10 was maximal at T_4 _and high levels were maintained until POD2. In the CAS group, IL-10 and IL-6 levels were undetected in most cases. Plasma levels of CRP were measured at T_1_, T_4_, POD1, and POD2. CRP levels started to increase at POD1 and were further increased at POD2 in both groups, but the increments were significantly higher in AAS patients than in CAS patients (Figure 5). Similar patterns were also found in PCT levels, with the difference that the maximal value was reached at POD1 (Figure 5). Similarly, there was a transient increase in leukocyte count at POD1 in AAS patients only, with values going back to normal at POD2 (data not shown). Plasma cortisol levels were not significantly different between AAS patients (111 ± 9 ng/ml) and CAS patients (105 ± 11 ng/ml) at T_1_. Both groups of patients showed a significant increase in plasma levels of cortisol at POD1 (AAS: 229 ± 44 ng/ml and CAS: 157 ± 26 ng/ml), with a tendency towards higher values for AAS patients (*P *= 0.07).

### Correlation between the levels of circulating NOD2 agonist and other parameters

The survey indicated that the inflammatory response took place after bacterial NOD2 agonist translocation. Among AAS patients, levels of circulating NOD2 agonist at T_4 _positively correlated with those of IL-10 at T_4 _and of cortisol at POD1 (r = 0.46, *P *= 0.04; and r = 0.55, *P *= 0.01, respectively). Interestingly, in AAS patients but not in CAS patients, cortisol levels at POD1 correlated with those of IL-10 at T_4 _and of PCT at POD1 (r = 0.59, *P *= 0.006; and r = 0.55, *P *= 0.02, respectively), and with the length of clamping (r = 0.45, *P *= 0.05). In AAS patients, the length of clamping correlated with levels of IL-6 at T_4 _and of PCT at POD1 (r = 0.53, *P *= 0.03; and r = 0.52, *P *= 0.02, respectively). There was no correlation between the length of clamping and levels of NOD2 agonist in the plasma. This is consistent with the results of Figure 2, showing that NOD2 agonist translocation occurred as soon as the gut was manipulated and reclinated by the surgeon, and thus before vascular clamping.

## Discussion

Derangement in gut barrier function occurs in many clinical settings. Endotoxin translocation has been evidenced in some cases and more frequently than in systemic bacterial translocation. Still, endotoxin is only representative of Gram-negative bacteria, microbial translocation may occur more regularly than previously reported after endotoxin measurement. The aim of this study was: to develop a tool allowing us to measure PGN, representative of both Gram-negative and Gram-positive; to address its presence within the bloodstream of patients in a clinical situation known to favor translocation of microbial products; to perform a survey during and after surgery to assess the frequency of the translocation of microbial products; and to attempt linking PGN translocation with the inflammatory process.

AAS is thought to be associated with endotoxin and bacterial translocation through the gut barrier, following manipulation of the gut by the surgeon and aortic clamping. However, evidence to prove this link was difficult to gather, probably because bacteria translocated into the bloodstream are rapidly killed and do not give rise to positive hemocultures [30,34]. Several studies, including ours, aimed to address this question by measuring circulating endotoxin [8,9]. However, this approach is hindered by the presence of many interfering or blocking molecules (soluble CD14, LPS-binding protein, lipoproteins) [15,16,35]. Thus the determination of LPS levels in the plasma of patients after surgery was not reliable enough, and did not allow for the demonstration that translocation was taking place systematically.

In the present study, we set up a new method for detecting bacterial NOD2 agonist in plasma, using a cell line transfected with NOD2, a general sensor of PGN through its minimal motif MDP [20,21], and a reporter gene under the control of the NF-κB transcription factor. Measurement of PGN is relevant in many aspects. First, it is the major component of Gram-positive bacteria and is also found in Gram-negative bacteria. Thus, when LPS detection addresses only Gram-negative bacteria, PGN detection addresses both types. Second, we showed that our system efficiently detected anaerobic bacterial PGN, expected to be more representative of the intestinal flora. Finally, the specificity of our test was confirmed by the use of a frameshift mutant of NOD2, which cannot be activated by bacterial PGN or its fragment [20,21]. This system showed that translocation of pathogen-associated molecular patterns (PAMPs) occurred in abdominal aortic surgery with a frequency higher than that measured so far, and that the assay of NOD2 agonist in plasma is a useful and sensitive tool for early detection of a bacterial product in the bloodstream. As the exact nature of circulating PGN is unknown, we used the terms 'NOD2 agonist' to name the circulating PAMP found in patients' plasma. The circulating bacterial material is probably closer to PGN than MDP. Indeed, in the HEK239T test, similarly to biochemically purified PGN, plasma samples led to a positive signal when added after the transfection step, in contrast to MDP, which had to be added in the presence of the transfection reagent in order to be detected. This is the first time that the NOD2 agonist was measured and found in AAS patients. Its detection is more reliable than that of LPS. Our results concur with those of a study of hemorrhagic shock in rat, showing that 30% had detectable amounts of LPS, but 73% were positive for circulating NOD2 agonist [30].

On the other hand, CAS patients (our negative control) did not show peaks for circulating NOD2 agonist or endotoxin, and had a relatively lower inflammatory response after the surgery, even if in some rare cases NOD2 agonist was detected in their plasma. Of course the CAS group underwent a shorter and less severe insult (shorter duration of surgery, less blood loss, rare blood transfusion), which may account for the lower inflammatory response on POD1 and POD2. Nevertheless, CAS patients were relevant as a control group for AAS patients. The groups were comparable in terms of weight, sex ratio, and pathology prior to surgery (atherosclerosis, diabetes, smoking habits). They were also undergoing vascular surgery, but without intestinal manipulation for CAS patients. We concluded that bacterial translocation is indeed tightly linked to reclination of the gut during surgery, in agreement with our previous observation [9]. Indeed, laparatomy and handing are sufficient to induce degradation in the intestinal brush border membrane [36].

Regarding medications, especially for statins, the CAS patients were higher consumers than AAS patients, although the difference was not statistically significant. Statins are known to have pleiotropic effects such as a reduction in inflammatory response, stabilization of atheroscleortic plaques, and improvement in vascular endothelial function, as well as a lipid lowering effect [37-39]. In our study, statin use had no significant effect on the levels of NOD2 agonist and inflammatory markers in either group. These observations suggest that differences in inflammatory response or circulating NOD2 agonist between the two surgery groups during the observational period were not related to statin use.

As expected, levels of inflammatory markers were higher in AAS than in CAS patients. In AAS patients, all endogenous markers of inflammation increased after circulating NOD2 agonist appeared. From these results, we assumed that bacterial translocation, which occurs before aortic clamping following abdomen incision and gut manipulation, may also contribute to a systemic inflammatory response in AAS patients. As illustrated by some correlations between NOD2 agonist and inflammatory markers (cortisol, IL-10), the presence of circulating NOD2 agonist contributes to the inflammatory response that was associated with a higher number of postoperative complications in AAS patients. Indeed, levels of IL-10 and cortisol are known to correlate with disease severity [8,40,41]. Furthermore, it is worth mentioning that PGN and PGN-derived structures are known to prime cells and enhance a further response to LPS [42], to synergize with cytokines [43], TREM-1 ligand [44], and other PAMPs [45] to favor inflammation, and to induce shock and organ dysfunction *in vivo *[46,47].

## Conclusions

In numerous clinical settings, microbial products derived from the gut are thought to worsen the disease. However, systemic bacterial translocation was rarely proven and rather endotoxin translocation has been measured. Endotoxins are only derived from Gram-negative bacteria and measuring PGN, which derives from both Gram-negative and Gram-positive bacteria could be more accurate. We report that in patients undergoing abdominal aortic surgery, most patients were positive for circulating NOD2 agonist, indicating that microbial translocation can be more frequent than previously thought when circulating endotoxin was measured. Furthermore, our data suggest that the presence of NOD2 agonist also contributes to the inflammatory response. Although the present test remains time consuming, we expect in the near future to develop a stable permanent transfected cell line that would allow us to perform the test within six hours. Furthermore, our results suggest that any other strategies that would be aimed to recognize the presence of PGN-like structure could be very useful.

## Key messages

• We developed a test able to detect PGN-like (NOD2 agonist) structure in plasma

• Gut manipulation is sufficient to induce translocation of microbial products

• Detection of NOD2 agonist is more reliable to detect translocation than measurement of endotoxin

• Among abdominal aortic surgery patients, levels of circulating NOD2 agonist positively correlated with that of cortisol and IL-10

## Abbreviations

AAS: abdominal aortic surgery; CAS: carotid artery surgery; CRP: C-reactive protein; ELISA: enzyme linked immunosorbent assay; fsNOD2: a frameshift mutant of NOD2; HEK: human embryonic kidney; IL: interleukin; LAL: limulus amebocyte lysate; LPS: lipopolysaccharide; MDP: muramyldipeptide; NF: nuclear factor; NOD: nucleotide-binding oligomerization domain; PAMPs: pathogen-associated molecular patterns; PBMC: peripheral blood mononuclear cells; PCT: procalcitonin; PGN: peptidoglycan; POD: postoperative day; SD: standard deviation; SEM: standard error of the mean; SIRS: systemic inflammatory response syndrome; TNF: tumor necrosis factor.

## Competing interests

The authors declare that they have no competing interests.

## Authors' contributions

OYK performed the measurements, analyzed the raw data, performed statistical analysis, drafted and contributed to the writing of the paper. AM included patients, collected the clinical information, performed statistical analysis and contributed to the writing of the paper. MB included patients, collected the clinical information and approved the manuscript. JMC designed the study, analyzed the raw data and contributed to the writing of the paper. PC designed the study and approved the manuscript. MAC designed the study, analyzed the raw data, drafted and contributed to the writing of the paper.
